# Promotion of malignant phenotype after disruption of the three-dimensional structure of cultured spheroids from colorectal cancer

**DOI:** 10.18632/oncotarget.24641

**Published:** 2018-03-23

**Authors:** Jose M. Piulats, Jumpei Kondo, Hiroko Endo, Hiromasa Ono, Takeshi Hagihara, Hiroaki Okuyama, Yasuko Nishizawa, Yasuhiko Tomita, Masayuki Ohue, Kouki Okita, Hidejiro Oyama, Hidemasa Bono, Takashi Masuko, Masahiro Inoue

**Affiliations:** ^1^ Department of Biochemistry, Osaka International Cancer Institute, Chuo-ku, Osaka, Japan; ^2^ Pathology, Osaka International Cancer Institute, Chuo-ku, Osaka, Japan; ^3^ Surgery, Osaka International Cancer Institute, Chuo-ku, Osaka, Japan; ^4^ Database Center for Life Science (DBCLS), Research Organization of Information and Systems (ROIS), Mishima, Shizuoka, Japan; ^5^ Cell Biology Laboratory, Department of Pharmaceutical Sciences, School of Pharmacy, Kinki University, Higashiōsaka, Osaka, Japan; ^6^ Current Affiliation: Department of Medical Oncology, Institut Català d’Oncologia, Barcelona, Spain

**Keywords:** spheroids, stemness, WNT, differentiated adenocarcinoma, colorectal cancer

## Abstract

Individual and small clusters of cancer cells may detach from the edges of a main tumor and invade vessels, which can act as the origin of metastasis; however, the mechanism for this phenomenon is not well understood. Using cancer tissue-originated spheroids, we studied whether disturbing the 3D architecture of cancer spheroids can provoke the reformation process and progression of malignancy. We developed a mechanical disruption method to achieve homogenous disruption of the spheroids while maintaining cell–cell contact. After the disruption, 9 spheroid lines from 9 patient samples reformed within a few hours, and 3 of the 9 lines exhibited accelerated spheroid growth. Marker expression, spheroid forming capacity, and tumorigenesis indicated that stemness increased after spheroid disruption. In addition, the spheroid forming capacity increased in 6 of 11 spheroid lines. The disruption signature determined by gene expression profiling supported the incidence of remodeling and predicted the prognosis of patients with colorectal cancer. Furthermore, WNT and HER3 signaling were increased in the reformed spheroids, and suppression of these signaling pathways attenuated the increased proliferation and stemness after the disruption. Overall, the disruption and subsequent reformation of cancer spheroids promoted malignancy-related phenotypes through the activation of the WNT and ERBB pathways.

## INTRODUCTION

Metastasis can originate from single cells, which can then invade into the circulatory systems through an epithelial–mesenchymal transition and form metastatic foci through a mesenchymal–epithelial transition in the distant organs [1]. On the other hand, it is also highly possible that cell clusters are the origin of metastasis, since cancer cell clusters inside microvessels are diagnosed as microvessel invasion during histopathological examinations, and a high risk of metastasis has been reported when circulating tumor cell clusters are detected in the blood [2]. The cell clusters can originate from fragments directly detached from the main tumor that had invaded into microvessels by collective cell migration [3]. However, consequences of disrupting the 3D structures of cancer have not been well studied due to the lack of experimental models. Conventional 2D cultures of established cell lines, which mostly have poorly differentiated characteristics, do not allow for the study of the 3D architecture of patient tumors.

We recently developed a method for the preparation and culture of primary cancer cells from various types of cancer [4–7]. The cancer tissue-originated spheroid (CTOS) preparation method is based on the principle that cell–cell contact must be maintained throughout the preparation and culture of the cancer cell clusters. CTOSs comprise only of cancer cells and are quite stable as long as the cell–cell contact is maintained. Furthermore, apico-basal polarity is preserved in CTOSs derived from differentiated adenocarcinoma of the colon [4]. Thus, CTOSs provide a model system for studying the 3D characteristics of cancer *in vitro*.

Stepwise accumulation of somatic alterations of genes can lead to colorectal cancer (CRC) [8]. The initial and critical event is the inactivation of the adenomatous polyposis coli (APC) tumor suppressor gene or activation of the ß-catenin oncogene (CTNNB1) [9]. APC is a negative regulator of WNT signaling, and mutational inactivation leads to the accumulation of ß-catenin in the nucleus and subsequent transcriptional activation of WNT target genes [10]. Although these findings imply that the WNT pathway plays critical roles in CRC, only a subset of cells in patient CRC samples show the accumulation of nuclear ß-catenin, even in CRCs with *APC* or *CTNNB1* mutations [11]. Some reports attribute this paradox to myofibroblast-secreted factors [12] or the activation status of the Ras/MAPK pathway [13]. However, the mechanisms underlying the substantial heterogeneity of nuclear ß-catenin accumulation in CRC tissues are not completely understood.

We reported previously that CTOS formation is a dynamic process [4]. CTOSs from CRC are spheroidal with smooth surfaces, and they are formed rapidly from fragments of cancer cell clusters within several hours [4]. Furthermore, CTOSs can be passaged *in vitro*, and occasional mechanical disruption is necessary for culture maintenance [4]. In this study, we investigated the mechanisms underlying the proliferation and stem-like properties of CRC CTOSs after disruption and reformation.

## RESULTS

### Disruption and reformation promote C45 CTOS growth

**Figure 1:**
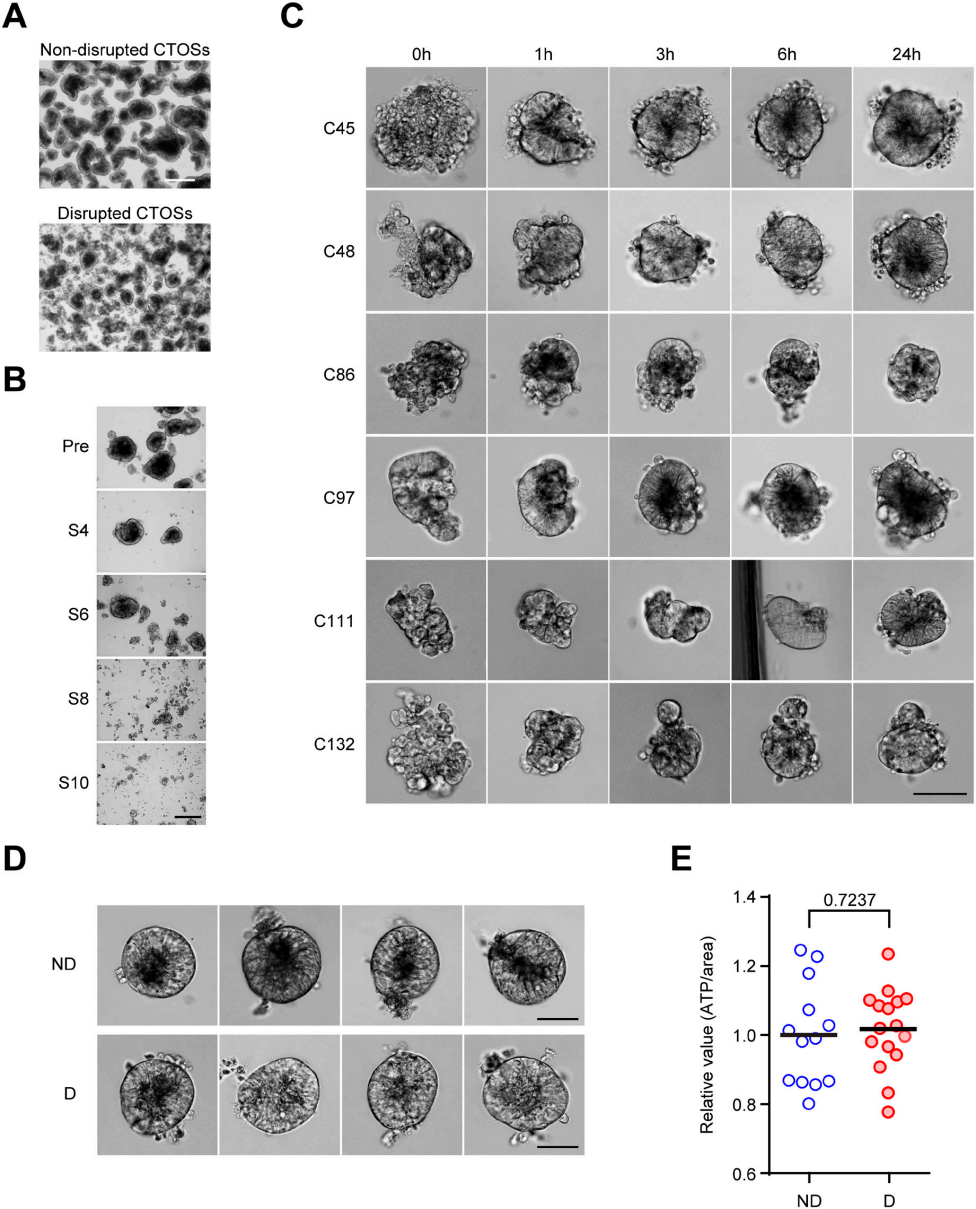
Reformation of cancer tissue-originated spheroid (CTOS) after mechanical disruption **(A)** Phase contrast images of C45 CTOSs before and after mechanical disruption. Scale bar, 500 μm. Representative images from more than 3 experiments are shown. **(B)** Optimization for the mechanical disruption of CTOSs. Phase contrast images show C45 CTOSs immediately after mechanical disruption with a 27G needle. The number of strokes (i.e. the number of passages through the needle) is indicated under the panels on the right. Scale bar, 100μm. Representative images from more than 3 experiments. **(C)** Phase contrast images show the time course of reformation of various CTOS lines. The time after disruption is indicated above the photos. Scale bar, 100 μm. Representative images from 24 CTOSs for each CTOS line. **(D)** Phase contrast images show the representative 4 CTOSs of C45 used in the disruption/reformation experiments. Twenty-four hours after disruption, the disrupted and non-disrupted CTOSs appeared to be identical. Scale bar, 50 μm. ND, non-disrupted; D, disrupted. **(E)** Intracellular ATP levels of the C45 CTOSs after 24 hours of disruption, approximately 100 μm in diameter, selected for non-disrupted (n = 13) and disrupted (n = 16) CTOSs. *P* value (unpaired t-test) is indicated.

To study the nature of cancer tissue fragments, which models the cell clusters that detached from the larger part of the tumor, we first optimized the CTOS mechanical disruption protocol so that the 3D architecture could be disrupted in a reproducible manner while cell–cell contact was maintained. We used C45, one of the CTOS lines, for the optimization experiments (Figure 1A). We passed CTOSs through a 27-gauge needle to disrupt them using shearing forces. With the optimized protocol (6 passages through the needle), CTOSs were disrupted into a sheet-like structure immediately after shearing (Figure 1B), and their smooth spheroidal shapes reformed within a few hours (Figure 1C), which was consistent with our previous observations [4]. Rapid reformation was commonly observed within several hours in CTOSs prepared from different patient CRC samples (Figure 1C). CTOSs that were undisrupted for more than a week and 24 h after disruption with similar sizes and shapes were morphologically indistinguishable from each other (Figure 1D, ND and D, respectively). The ATP levels, which reflect the number of metabolically active cells, were comparable between non-disrupted and disrupted C45 CTOSs with similar sizes (Figure 1E).

**Figure 2:**
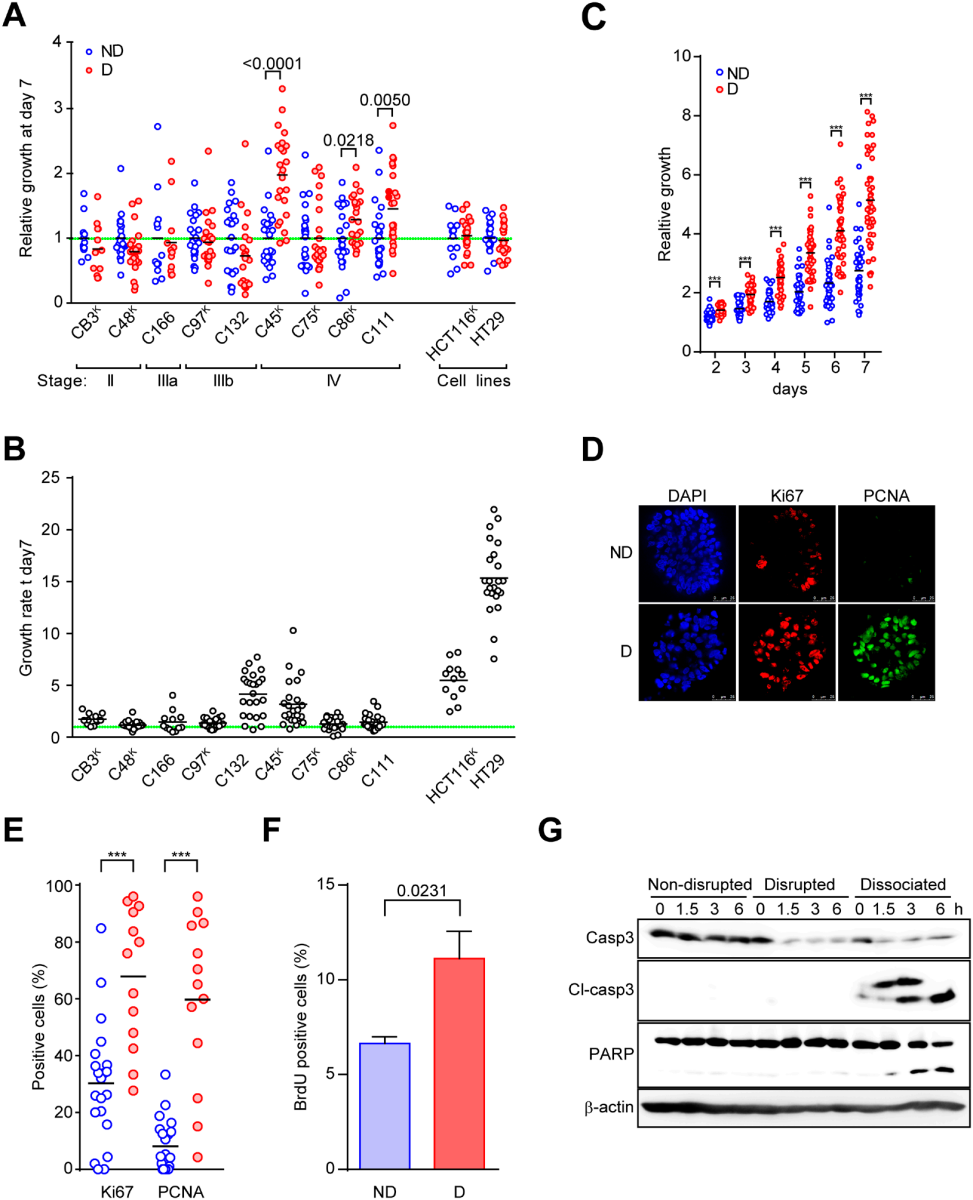
Disruption and reformation promote C45 CTOS growth **(A)** Growth of various CTOSs derived from patient tumors and spheroids from two cell lines. The clinical stage is indicated. The superscript ‘K’ indicates a KRAS mutant (also see Figure 3B). The ATP value on day 7 was adjusted by the area of each CTOS on day 0. The values relative to the average values of non-disrupted CTOSs are shown for each CTOS. A value of 1 is indicated by the green dotted line. Each bar represents an average. *P* values are indicated. N = 12 (CB3), 24 (C48), 12 (C166), 24 (C97), 24 (C132), 24 (C45), 24 (C75), 23 (C86), 24 (C111), 12 (HCT116), 23 (HT29). **(B)** The growth of various non-disrupted CTOSs and spheroids from two cell lines used in Figure 2A. The growth rate was determined as the spheroid area on day 7 divided by the area on day 0. **(C)** Time course of the growth of disrupted C45 CTOS (n = 47) versus non-disrupted CTOS (n=43). The graph shows the CTOS area at each time point relative to the area on day 0. Each dot (non-disrupted (ND), blue; disrupted (D), red) represents one CTOS. Each bar represents the average. ^***^P<0.0001. **(D)** Representative images of immunofluorescence staining of Ki67 or PCNA. **(E)** The percentage of positive cells stained with Ki67 or PCNA in disrupted (n = 13) and non-disrupted (n = 19) CTOSs. Each bar represents the average. ^***^P<0.0001. Results are representative from 3 independent experiments. **(F)** Changes in BrdU incorporation assessed by flow cytometry of CTOSs after disruption are shown as the averages and standard deviations of four experiments. Means ± SEM are shown. *P* value (unpaired t-test) is indicated. Results are representative from 3 independent experiments. **(G)** Western blotting with the indicated antibodies demonstrates that apoptosis occurred in dissociated CTOS cells after C45 CTOS disruption. The time points are indicated above the panels. Cl-caspase 3, cleaved caspase 3. For dissociation, the CTOSs were dispersed into single cells using trypsin treatment. Results are representative from 3 independent experiments.

Next, to evaluate the effect of disruption on the cancer cells, we investigated the growth of CTOSs after mechanical disruption. Three of 9 CTOSs that were derived from different patient tumors, including C45 CTOSs, showed significantly increased growth rates after disruption (Figure 2A). The three CTOSs (C45, C86, and C111) that showed growth increases were all from patients with stage IV cancers. Neither of the two CRC cell lines that we tested showed changes in their growth rates after disruption under 3D culture conditions. The increase in growth after disruption was not related to the *KRAS* status (Figure 2A) or basal growth rate (Figure 2B). The differences in size were significant by day 2, and disrupted CTOSs had approximately doubled in size after 7 days when compared with non-disrupted CTOSs (Figure 2C). At 24 h after disruption, nuclear PCNA and Ki67 positivity (Figure 2D, 2E) as well as BrdU incorporation (Figure 2F) had increased significantly. CRC cells that were completely dissociated into single cells underwent robust apoptosis [4] (Figure 2G). This is compatible with our previous finding that approximately 50% of the single cells after 6 hours of dissociation from CTOSs undergo anoikis, a distinct type of apoptosis induced by the loss of E-cadherin mediated cell-cell interactions [4]. Notably, apoptosis was not observed in the disrupted CTOSs or in the non-disrupted CTOSs (Figure 2G). These results indicate that the disruption of cancer cell cluster stimulates proliferation in a subset of CRC CTOSs.

### Disruption and reformation promote stemness in CTOSs

**Figure 3:**
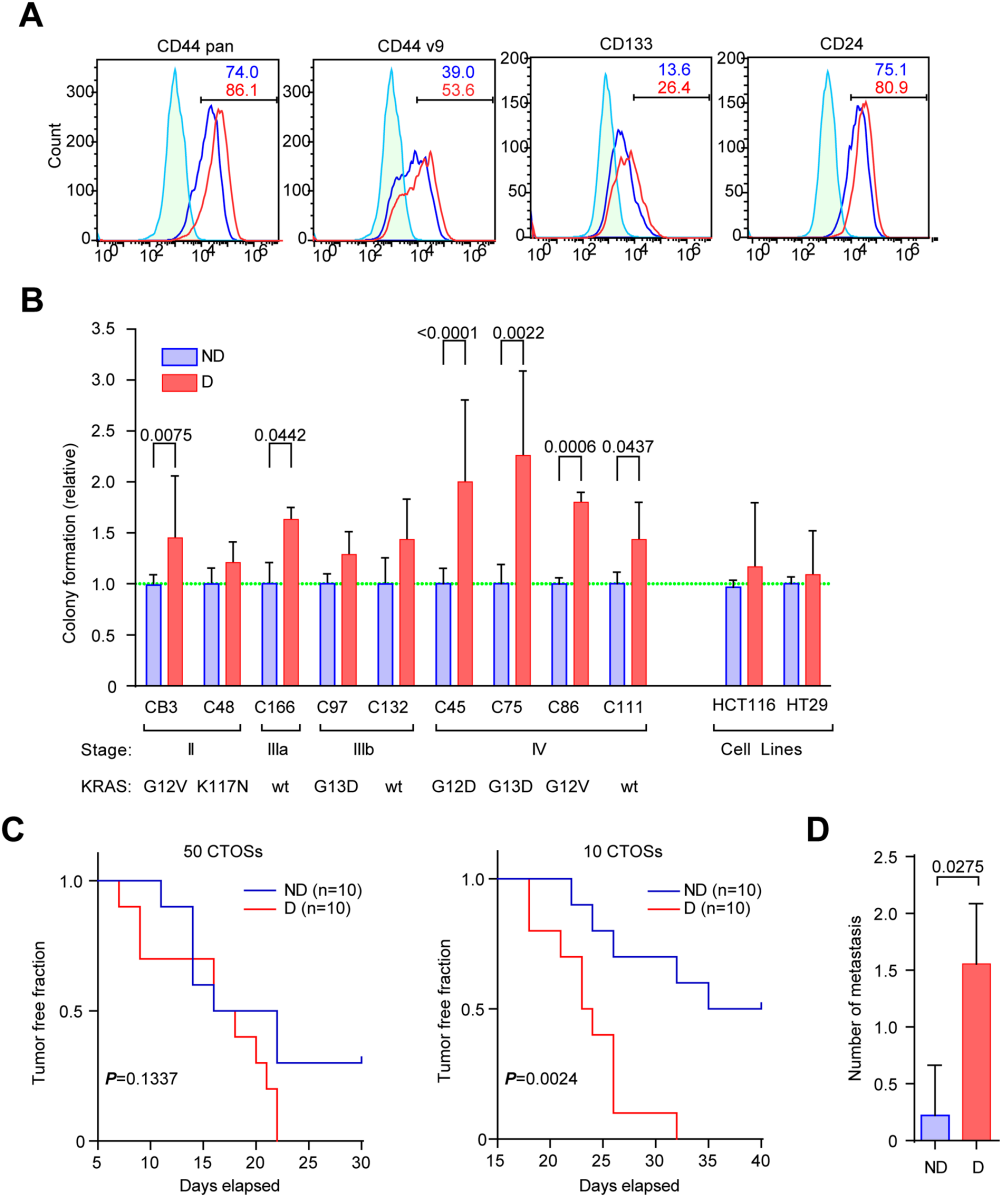
Disruption and reformation promote stemness in CTOSs **(A)** FACS analysis using antibodies for the indicated markers. Green line, isotype control; blue line, non-disrupted C45 CTOSs; red line, disrupted C45 CTOSs. The percentage of cells in the indicated gates is shown. Results are representative from 4 independent experiments. **(B)** The spheroid-forming capacity of single cells from various CTOSs derived from patient tumors and spheroids from two cell lines. The values shown are relative to non-disrupted CTOSs. A value of 1 is indicated by the green dotted line. Means ± SEM are shown. *P* values are indicated. N = CB3, 20; C48, 6; C166, 3; C97, 4; C132, 6; C45, 24; C75, 3; C86, 3; C111, 6; HCT116, 24; HT29, 24. **(C)** Kaplan-Meier analysis of tumor formation after the C45 CTOSs were injected subcutaneously. The number of CTOSs injected (50 or 10) is indicated above the graphs. ND, non-disrupted, n = 10; D, disrupted, n = 10; *P* values of log-rank test are indicated. Tumor formation was judged by palpation. Results are from 2 independent experiments. **(D)** The number of liver metastases 28 days after the C45 CTOSs were injected into the mouse spleens. Means ± SEM are shown. N = 9, *P* value (unpaired t-test) is indicated.

To examine whether the disruption of CTOSs have another effect on the malignancy of CRC cells other than proliferation, we next evaluated the cancer stem-like characteristics of disrupted CTOSs. The number of cells expressing pan-CD44, CD44v9, CD133, and CD24, all of which were reported to be markers of cancer stem-like cells in CRC [14–16], increased after disruption of C45 CTOSs (Figure 3A). The sphere-forming capacity from single cells is one of the hallmarks of stemness [14], which reflects the capacity of self-renewal, proliferation and resistance to anoikis [17–19]. The sphere-forming capacity was increased in 6 out of 9 CTOSs, including C45, after the disruption, whereas the sphere-forming capacity was unchanged in two cell lines (Figure 3B). Notably, 4 out of 4 CTOSs from stage IV had increased spheroid-forming capacity.

Tumorigenicity in immunodeficient mice is another hallmark of stemness in cancer cells [20]. Subcutaneous injection of disrupted C45 CTOSs into nude mice was more efficient than non-disrupted CTOSs in generating tumors (Figure 3C). When disrupted CTOSs were injected into the spleens of nude mice, the number of liver metastases increased when compared with non-disrupted CTOSs (Figure 3D). These results suggest that disruption and reformation of the CTOSs promote stemness.

### Changes in gene expression during CTOS disruption/reformation correlate with prognosis of patients with CRC

**Figure 4:**
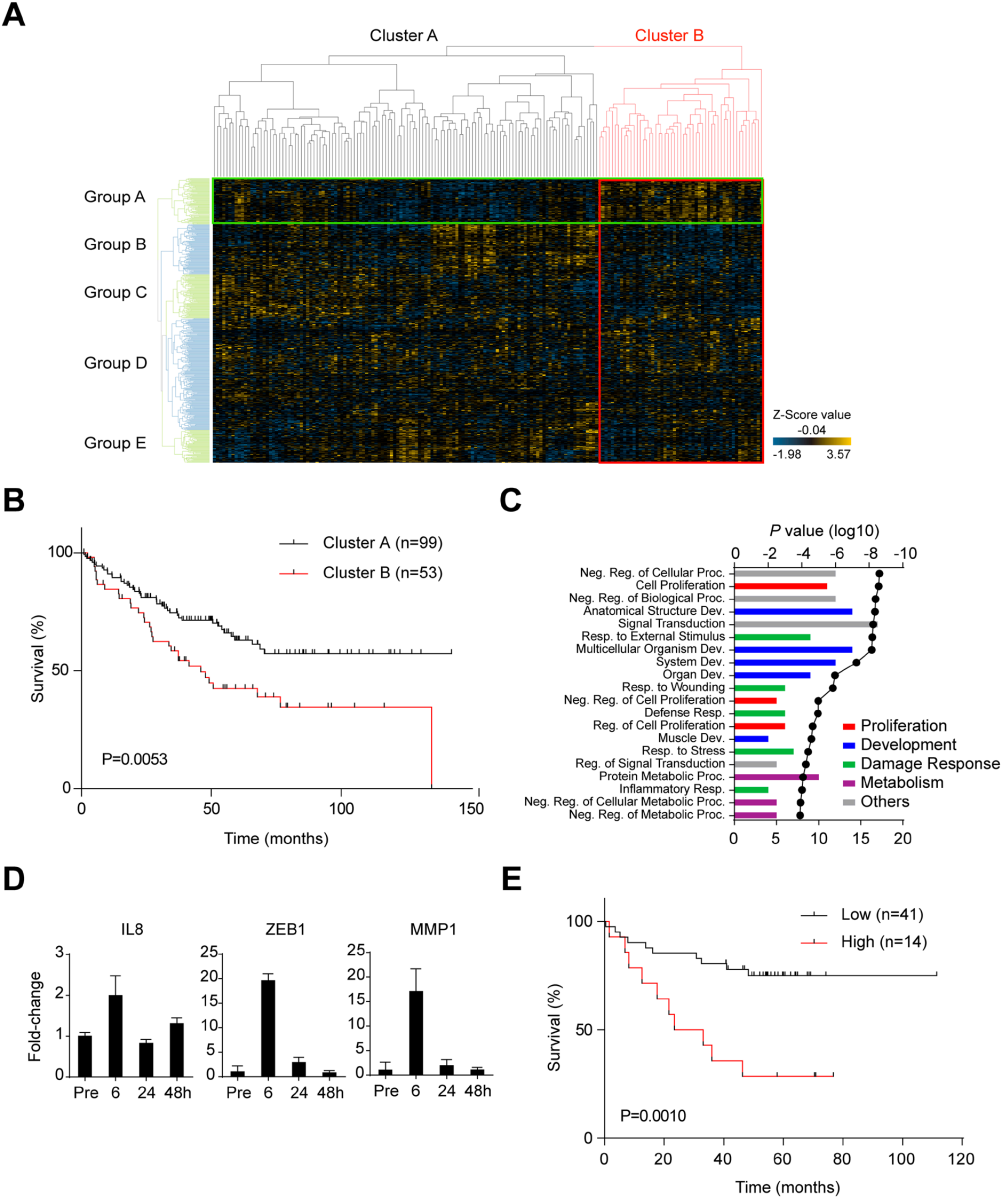
Changes in gene expression during the disruption/reformation process in C45 CTOSs **(A)** Clustering analysis of microarray data using the disruption/reformation-related gene set, which comprised of genes that were upregulated 6 and 24 h after CTOS disruption. The data set is from GSE17536, and a heat map of the cluster analysis is shown. The spots classified into cluster B are bordered by a red line, while those classified into group A are bordered by a green line. **(B)** Kaplan-Meier analysis of the patient’s overall survival (GSE17536) according to cluster (Figure 4A). *P* = 0.0053 (log-rank test). **(C)** The top twenty gene ontology (GO) terms associated with the disruption signature (85 genes); the lowest *P* values are shown, and the GO terms are ranked by *P* value. The categories of the terms are indicated by color. The line is the log10 of the *P* value. The bars show the number of genes that were common to both the GO term’s gene set and the disruption signature. Abbreviations are Reg., regulation; Proc., process; Neg., negative; Dev., development; Resp., response. **(D)** Time course of gene expression in C45 CTOS before (Pre) and after disruption at the indicated time points as determined using real time PCR analysis. Error bars indicate SEM. *P* <0.0001 (one-way ANOVA) in all of the gene expression. **(E)** Kaplan-Meier analysis of the patient’s overall survival (GSE17537) according to signature group. *P* = 0.0010 (log-rank test).

Since both proliferation and cancer stem-like cell properties are responsible for malignant feature of CRCs, we investigated whether the characteristics of disrupted CTOSs were linked to poorer prognosis of CRCs using a bioinformatics approach. Transcriptome profiling was performed on non-disrupted and disrupted C45 CTOSs at 6 and 24 h after disruption. A total of 655 genes were upregulated and 516 were downregulated at both 6 and 24 h after disruption. We defined the 655 upregulated genes as the “disruption/reformation-related gene set.” Next, we applied this gene set to a cluster analysis on a human CRC array dataset (GSE17536). Clustering showed two distinctive classes of patients with CRC (Figure 4A, Cluster A and B) with significantly different prognoses (Figure 4B). Meanwhile, the disruption/reformation-related genes were clustered into 5 groups (Figure 4A, Group A to E). The genes in Group A were upregulated in patients with a poorer prognosis (Cluster B) and downregulated in patients with a better prognosis (Cluster A) (Figure 4A). In addition, when Cluster A was subdivided into Cluster A1, A2 and A3 based on the dendrogram under original Cluster A, Cluster A1 had higher expression of Group A genes than Cluster A2 and A3 (Supplementary Figure 1A). Cluster A1 and B, both of which have high expression of Group A genes, had no significant difference in prognosis (Supplementary Figure 1B, *P*=0.267), while A3 had significantly better survival (Supplementary Figure 1B, *P*=0.014). Cluster A2 tended to have better survival than Cluster B, although without statistical significance (Supplementary Figure 1B, *P*=0.067). We called these 85 genes the “disruption signature” (Supplementary Table 1). Gene ontology (GO) analysis of the disruption signature genes showed an enrichment of proliferation and development gene sets (Figure 4C). The disruption signature contained the inflammation-related gene IL8, the epithelial–mesenchymal transition (EMT) genes ZEB1 and VIM, and the protease genes PLAU and MMP1. The alteration of these genes in disrupted CTOSs was confirmed by quantitative RT-PCR for IL8, ZEB1, and MMP1 (Figure 4D). To confirm the correlation between this disruption signature and CRC prognosis, we next applied the disruption signature to another CRC dataset with information on the clinical outcome (GSE17537). Cluster analysis in the new dataset showed that the disruption signature divided patients into two groups with a statistically significant difference both in disease free survival and overall survival; patients with tumors expressing higher levels of the disruption signature genes had a worse prognosis (Supplementary Figures 2, 3 and Figure 4E).

### Activation of WNT signaling is critical in the disruption/reformation process

**Figure 5:**
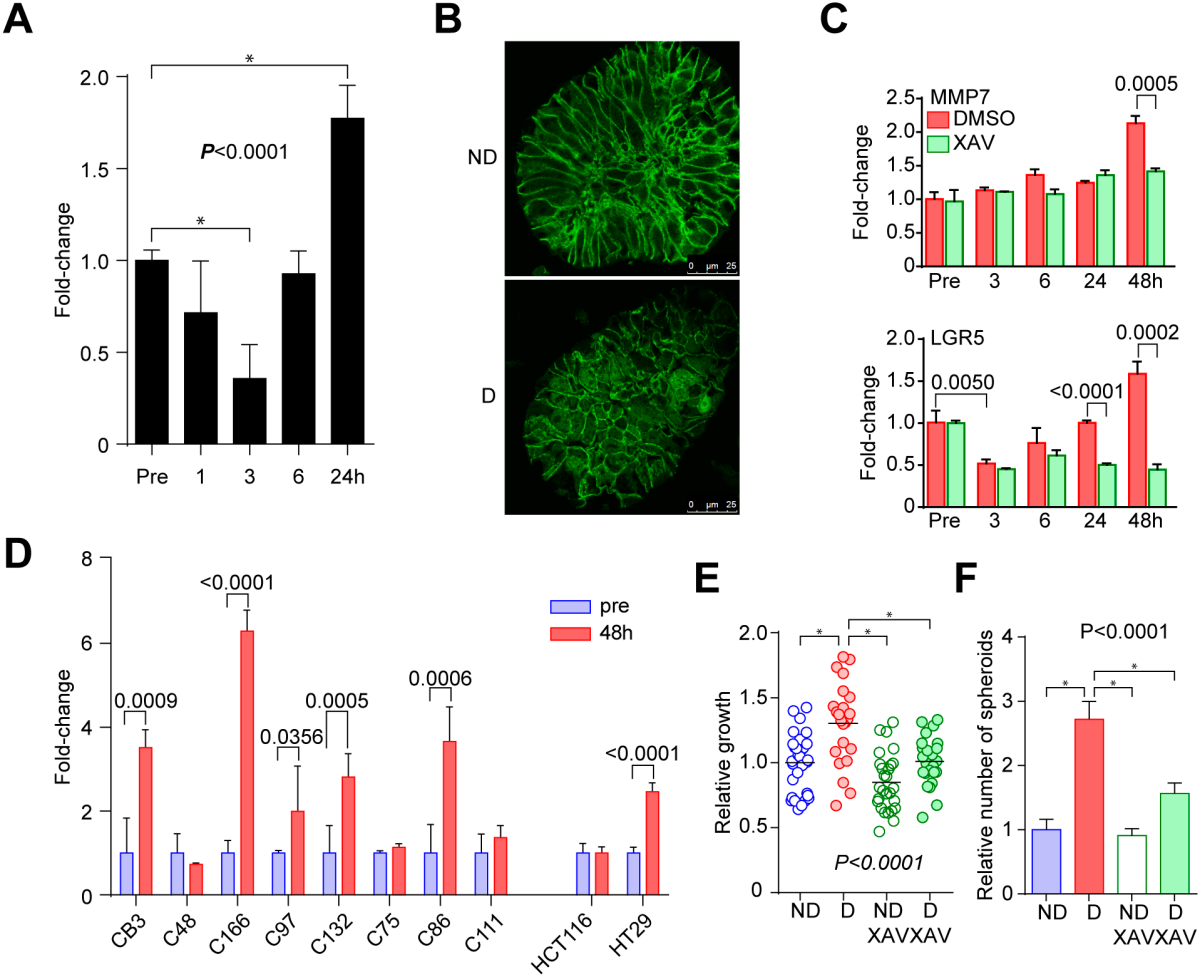
Activation of WNT signaling in the disruption/reformation process **(A)** Time course analysis of TCF/LEF promoter activity in C45 CTOSs expressing the TOPFlash vector. Error bars indicate SEM. *P* < 0.0001 (one-way ANOVA). ^*^Significance according to Tukey’s multiple comparison test. Results are representative from 5 independent experiments. **(B)** Immunohistochemistry of β-catenin in C45 CTOS. ND, non-disrupted, D, disrupted. Scale bar, 10 μm. Images are representative from more than 10 CTOSs of 3 independent experiments. **(C)** Real-time PCR analysis of MMP7 gene expression. C45 CTOSs were treated with DMSO or with 1 μM XAV939. Error bars indicate SEM. *P*<0.0001 for DMSO group, and *P* = 0.00005 for XAV group (one-way ANOVA). *P* values of unpaired t-test for DMSO and XAV treated CTOSs are shown. **(D)** Real-time PCR analysis of MMP7 gene expression 48 h after disruption in CTOSs derived from patient-derived tumors and in spheroids from two cell lines. Error bars indicate SEM. *P* values for pre- and 4 h after disruption in each CTOS and cell line are indicated. **(E)** The relative growth of C45 CTOSs 7 days after disruption. Disrupted CTOSs were untreated (n = 23) or treated (n = 24) with 1 μM XAV939. Non-disrupted CTOSs. *P* = 0.0002 (one-way ANOVA). ^*^Significance according to Tukey’s multiple comparison test. Results are from 3 independent experiments. **(F)** The relative number of spheroids formed from single C45 CTOS cells. Disrupted CTOSs were untreated or treated with 1 μM XAV939. Means ± SEM are shown. N = 3. *P* = 0.0003 (one-way ANOVA). ^*^Significance according to Tukey’s multiple comparison test. Results are representative from 3 independent experiments.

We next investigated WNT signaling since WNT signaling regulates multiple processes of development, and that the GO analysis revealed genes related to development were enriched in the disruption signature (Figure 4C). The TCF/LEF promoter reporter activity increased at 24 h in C45 CTOSs after a transient decrease immediately after disruption (Figure 5A), which suggested that WNT signaling was activated. We observed an increase in the nuclear localization of β-catenin 24 h after disruption of C45 CTOSs (Figure 5B). To evaluate the consequences of upregulated WNT signaling, we analyzed the microarray data of 6 and 24 hours after disruption. A list of 31 known WNT target genes in colorectal cancer was extracted from the report by Nusse et al. on their web site (Supplementary Table 2, https://web.stanford.edu/group/nusselab/cgi-bin/wnt/target_genes (last update: June 2017). Fifteen of these target genes exhibited >1.5-fold increase at either time-point, while only 3 were suppressed at both time-point (Supplementary Figure 4, Supplementary Table 2). The expressions of WNT target genes, MMP7 and LGR5, were upregulated 48 h after disruption of C45 CTOSs; the upregulation was suppressed by treatment with XAV939, a tankyrase inhibitor that inhibits the WNT pathway (Figure 5C). LGR5 expression transiently decreased immediately after disruption and increased at 48 h. MMP7 expression increased 48 h after disruption in 5 out of 8 additional CTOSs (Figure 5D). Thus 6 out of 9 (67%) cases including C45 showed increased MMP7 expression after disruption, and 4 of those 6 CTOSs had increased spheroid-forming capacity.

Next we assessed the functional role of WNT signaling in the disruption/reformation process using WNT pathway inhibitors. XAV939, a tankyrase inhibitor, suppressed the increases in growth and the capacity to form spheroids after disruption of C45 and C111 CTOSs (Figure 5E, 5F, Supplementary Figure 5). Pyrvinium, a casein kinase activator, also suppressed the spheroid formation and the growth of C45 CTOS (Supplementary Figure 6). Taken together, these findings indicated that the activation of the WNT pathway after mechanical disruption of CTOSs plays functional roles in both the growth and stemness in CTOSs.

### Activation of ERBB signaling is critical in the disruption/reformation process in C45 CTOS

**Figure 6:**
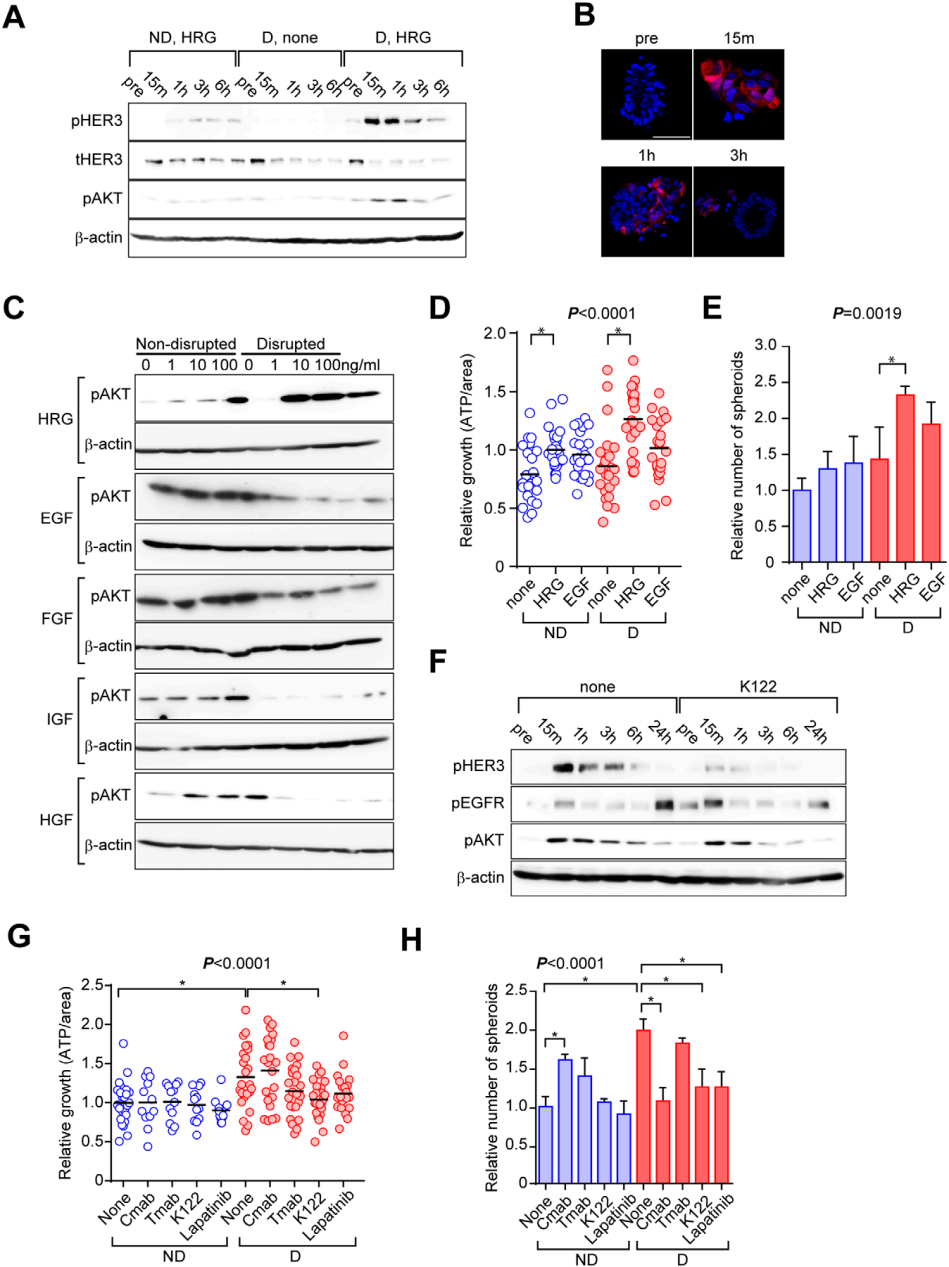
Activation of ERBB signaling is critical to the disruption/reformation process of C45 CTOSs **(A)** Western blotting of HER3/AKT signaling using the indicated antibodies at the indicated times. CTOSs were starved for 48 h and then disrupted either in the presence or absence of growth factors. Samples were collected before (pre) and after disruption. ND, non-disrupted; D, disrupted; HRG, 10 ng/ml heregulin β1; none, no growth factor added. Results are representative from 3 independent experiments. **(B)** Representative images of samples analyzed in (*A*) stained with the pAKT antibody from 2 independent experiments. **(C)** Western blotting showing the effect of various growth factors on AKT activation 15 min after CTOS disruption. CTOSs were starved for 48 h and then growth factors were added 24 h before disruption. Dose and antibodies are indicated. HRG, heregulin β1; EGF, epidermal growth factor; FGF, basic fibroblast growth factor; IGF, long-insulin growth factor-1; HGF, hepatocyte growth factor. Results are representative from 2 independent experiments. **(D)** Relative growth of C45 CTOSs. Disrupted CTOSs were treated with the indicated growth factors, 10 ng/ml. *P* < 0.001 (one-way ANOVA). ^*^Significance according to Tukey’s multiple comparison test. ND: n = none, 22; HRG, 24; EGF, 24. D: n = none, 23; HRG, 24; EGF, 23. Results are from 3 independent experiments. **(E)** The number of spheroids derived from single cells is shown as the value relative to non-disrupted CTOSs. Disrupted CTOSs were treated with the indicated growth factors, 10 ng/ml. *P* = 0.0019 (one-way ANOVA). N = 3. ^*^Significance according to Tukey’s multiple comparison test. Results are representative from 3 independent experiments. **(F)** Western blotting showing the effect of K122, a HER3 antibody, on intracellular signaling of C45 CTOSs cultured in StemPro medium. The antibodies and times relative to disruption are indicated. Results are representative from 3 independent experiments. **(G)** Relative growth of C45 CTOSs treated with the indicated antibodies or with lapatinib, 500 nM. Cmab, cetuximab; Tmab, trastuzumab, 10 μg/ml. N = 3. *P* < 0.0001 (one-way ANOVA). ^*^Significance according to Tukey’s multiple comparison test. ND: n = none, 24; Cmab, 12; Tmab, 12; K122, 12; Lapatinib, 12. D: n=none, 24; Cmab, 22; Tmab, 24; K122, 24; Lapatinib, 22. Results are from 5 independent experiments. **(H)** The relative number of spheroids derived from single C45 cells treated with the indicated antibodies, 10 ng/ml or with lapatinib. 500 nM. N = 3. *P* < 0.0001 (one-way ANOVA). ^*^Significance according to Tukey’s multiple comparison test. Results are representative from 5 independent experiments.

ERBB family signaling, especially through EGFR [21], HER2 [22], and HER3 [23], plays important roles in CRC. In addition, we previously reported that AKT activation downstream of HER3 plays important roles in the growth of CTOSs in lung and urothelial cancers [5, 6]. While disruption of CTOS did not affect AKT phosphorylation in the absence of any growth factors in the medium, supplementation with heregulin (HRG), a HER3 ligand, after disruption immediately increased phosphorylation of AKT (Figure 6A, 6B, 6C). Other ligands, including EGF, bFGF, IGF-1, and HGF, did not increase AKT phosphorylation in disrupted CTOS (Figure 6C). When stimulated with HRG, the growth of both non-disrupted and disrupted CTOS increased, while EGF had no significant effect (Figure 6D). As for the spheroid-forming capacity, HRG significantly increased the number of spheroids in disrupted CTOS but not in non-disrupted CTOS, while EGF had no significant effect (Figure 6E). K122, a neutralizing antibody of HER3, suppressed the increase of HER3 and AKT phosphorylations after CTOS disruption (Figure 6F). When treated with K122, increased CTOS growth after disruption was suppressed (Figure 6G). Cetuximab, an EGFR antibody, had no effect. Trastuzumab, a HER2 antibody, as well as lapatinib, a dual inhibitor of EGFR and HER2 kinase, attenuated the increase in growth, although this was not statistically significant (Figure 6G). K122, as well as cetuximab and lapatinib, suppressed the increase in the spheroid-forming capacity, while trastsuzumab had no effect (Figure 6H). Cetuximab increased the spheroid-forming capacity in non-disrupted CTOSs, but the underlying mechanism was not clear. Thus, ERBB signaling, especially HER3 signaling, is involved in C45 CTOS reformation and subsequent stimulation of growth and stemness after disruption.

## DISCUSSION

Most CRCs are differentiated adenocarcinomas in which tubular differentiation and glandular structures are maintained to some extent. Accordingly, it is not surprising that CRC cells retain the repair system found in normal intestinal epithelium. Although the repair of normal intestinal epithelium requires factors that are secreted by the surrounding non-epithelial cells [24–26], cancer cells are much less dependent on neighboring non-epithelial cells due to cancer cell autocrine/paracrine secretion or cell-autonomous activation of signal pathways. Several recent studies have shown that stemness can be induced in cancer cells [27, 28], and it has even been suggested that differentiated normal epithelial cells become stem cells after injury [29]. The recruitment of stem cells or progenitors is a critical step in wound repair of epithelial cells, and the induction of stemness during the disruption/reformation of CTOS is also related to the repair of normal epithelium.

In addition to fragmentation from the main tumor in the lesions of microvessels, disruption/reformation might also occur to various extents in patient tumors. We demonstrated here that the signature of gene expression after disruption was linked to CRC with poor prognosis, indicating that the *in vitro* findings in this study were not simply an artificial hallmark of the culture system. CRC often shows the infiltration of inflammatory cells, and chronic inflammation has been linked with the development of cancer [30, 31]. This might be due in part to the enhanced production of reactive oxygen species via the infiltrating immune cells [32, 33]. Exposure to such a destructive microenvironment results in a cycle of tissue damage and regeneration, and this could be similar to the disruption/reformation observed in CTOSs. Inflammatory cytokines, which promote malignancy [31, 34], were upregulated after mechanical disruption of CTOSs. On the other hand, when the cancer cells were not eradicated by therapeutic treatments such as chemo- or radiotherapy, loss of cell–cell contact due to death of neighboring cells may have effects similar to the effects of CTOS disruption. Tumor hypoxia due to anti-angiogenesis therapy can also disrupt 3D structures. Accordingly, the disruption/reformation process might be one cause of the therapy-induced malignant conversion of the tumor [35, 36]. The processes of disruption and subsequent reformation may be a cause, but not a mere consequence, of malignant progression.

Changes in oxygen levels after disruption of CTOSs might be a mechanism of subsequent events. Indeed, as we reported previously, a core region greater than 200 μm in diameter in the CTOSs can be hypoxic [4], resulting in decreases in growth [37]. To eliminate the possible effects of changes in oxygen levels inside the CTOSs, we selected smaller CTOSs (< 200 μm in diameter). The microarray data revealed that there were only minimal changes in the expression of hypoxia-inducible genes such as *CA9* and *GLUT1*. In addition, the proliferation of cells on the surfaces of CTOSs, which are supposed to be well oxygenated, was lower in non-disrupted than in disrupted CTOSs. Thus, changes in oxygen levels are unlikely to be the sole cause of the events that occur after disruption.

WNT signaling was activated after CTOS disruption, and inhibition of WNT signaling decreased CTOS growth and spheroid formation after disruption. The mechanism that leads to the activation of WNT signaling in the disruption/reformation CTOS model is not yet clear. WNT2B, a canonical activator of WNT signaling, was markedly upregulated according to the microarray data, although WNT inhibitors such as SFRP1 and DKK1 were also upregulated. This reflects the complexity of the WNT signaling pathway response to CTOS disruption and reformation. Loss of cell–cell contact after disrupting CTOSs can also trigger β-catenin translocation to the nucleus [38].

Here, we showed that the HRG-HER3-AKT axis is involved in the disruption/reformation process of a CRC CTOS line. Specifically, HRG treatment increased growth and stemness, and HER3 inhibition suppressed both growth and stemness in C45 CTOSs (Figure 7). The breakdown of ligand–receptor segregation is one possible mechanism for HER3 activation after mechanical injury to the epithelium [39]. The importance of HER3 in CRC progression was demonstrated previously by clinicopathological analyses [40, 41] as well as by experiments using conventional 2D culture of CRC cell lines [40]. This study posits that HER3 plays another role in CRC malignancy in the context of 3D conformation, at least in some of the cases.

## MATERIALS AND METHODS

### CTOS preparation and cell culture

The collection, handling, and use of human tumor tissue samples were conducted in accordance with protocols approved by the institutional ethics committees at the Osaka Medical Center for Cancer and Cardiovascular Diseases. The detailed methods for CTOS preparation and expansion were described previously [4]. CTOSs were cultured in suspension in StemPro hESC SFM (Life Technologies, Carlsbad, CA). Every 2–3 days, the medium was partially replaced and the CTOSs were cultured in suspension until used in experiments. To assess the effects of growth factors, CTOSs were cultured in basal medium containing the following growth factors: HRG (heregulin), β1/neuregulin 1 (PeproTech, Rocky Hill, NJ, 100-03), long-IGF1 (GroPep, Thebarton SA, Australia, BU020), bFGF (Life Technologies, 13256029), EGF (Life Technologies, PHG0311L), and HGF (R&D Systems, Minneapolis, MN, 294-HGN-005). Basal medium consisted of DMEM/F12, 1x GlutaMAX, 0.1 mM 2-mercaptoethanol, and 2% BSA.

### Mechanical disruption of CTOSs

We used a 27-gauge needle to shear CTOSs, raising and lowering the plunger several times. We prepared fresh CTOSs from mouse xenografts and cultured them for 7 days *in vitro* to avoid the possible effects of disruption during the preparation of CTOS from the xenografts. The CTOSs were filtered, CTOSs >100 μm in diameter were collected, and the CTOS suspension was then divided into two groups. In the disrupted group, the CTOS were disrupted by needle shearing. In the non-disrupted group, CTOSs were cultured without disruption.

### Cell line experiments

HCT116 and HT29 cells were obtained from American Type Culture Collection (ATCC). Spheroids were generated from the cell lines by suspending the cells in StemPro-FCeM (Nissan Chemical Industries, Tokyo, Japan) to avoid spheroid fusion, and then culturing them in suspension for about 10 days. The cultures were filtered through a 100 μm filter, and spheroids >100 μm in diameter were collected. The spheroids were disrupted by 20 strokes through a 27-gauge needle for HT29 or 4 strokes for HCT116 and then cultured for one more day.

### CTOS growth assay

After disruption, CTOSs were cultured for one additional day, and the CTOSs that were similar in size and shape were selected and plated in 96-well plates at a density of one CTOS per well (n = 24) for each group, and photographed under a microscope to measure the area projected onto a X-Y plane (day 0). Seven days after disruption, photographs were taken and intracellular ATP levels were measured by Cell Titer Glo (Promega), and the value was adjusted by the area from day 0 for each CTOS.

### Spheroid forming assay

After disruption, CTOSs were cultured for one additional day, and both disrupted and non-disrupted control CTOSs were dissociated into single cells by 0.25% trypsin/EDTA treatment. The single cells were suspended and cultured in StemPro-FCeM (StemPro hESC 50x supplemented with FCeM-D/F) to avoid fusion of the spheroids. The cell suspensions (1 × 10^3^ per ml in 3 ml) were cultured in poly-HEMA- (Sigma-Aldrich, Tokyo, Japan) coated 6-well plates for 14 days. Six wells were analyzed for each experimental condition. On day 14, the number of spheroids with a size greater than 100 μm in diameter was counted.

### Preparation of rat monoclonal antibodies against HER3

Female F344 rats were administered subcutaneously and intraperitoneally with RH7777 rat hepatoma cells (generously provided by Tanabe Mitsubishi Pharm.) expressing human HER3 linked to GFP (RH7777-HER3-GFP cells) in the first immunization, followed by four booster intraperitoneal and intravenous injections of RH7777-HER3-GFP cells at a 10-day interval. Three days after the final immunization, spleen cells of the immunized rats were isolated and fused with P3×63Ag8.653 mouse myeloma cells in 50% polyethylene glycol 1540 (Roche Diagnostics, Tokyo, Japan). After the cell fusion, hybridoma cells were selected in 7% FBS-containing RPMI1640 medium (Sigma) supplemented with hypoxanthine, aminopterin, and thymidine (HAT supplement; Life Technologies, Tokyo, Japan). Antibodies secreted from hybridoma clones were selected for reactivity against HEK293-HER3-GFP cells in a GFP expression-dependent manner [42–44]. Anti-HER3 (K122/IgG2a, κ) mAb were also confirmed for no reactivity with RH7777 or HEK293 cells expressing HER1, HER2, or HER4 proteins.

### Reagents

For inhibition analysis, we used XAV939 (Selleck Chemicals), pyrvinium pomate (Sigma-Aldrich) and lapatinib (LC Laboratories). Cetuximab and trastutuzumab were provided by the institutional pharmacy. For immunohistochemistry, we used Ki67 (Leica Microsystems, Wetzler, Germany, NCL-Ki67p), phospho-AKT (p-AKT) (Ser473, 4060), and PCNA (2586), from Cell Signaling Technologies (CST, Danvers, MA). For western blotting, we used antibodies against β-actin (Sigma, AC-74), PARP (BD, 51-6639GR), and HER3 (NanoTools, 5A12). Caspase 3 (9662), cleaved caspase 3 (D175), p-HER3 (Y1289, 21D3), p-EGFR (Y1068, D7A5), and p-AKT (Ser473, D9E) were from CST. For flow cytometry, the BrdU (555627) and CD24 (555427) were from BD; CD133 (293C3) was from Miltenyi Biotec. The antibodies against pan-CD44 (IM7) and CD44v9 were generated by T.M. [45].

### TOPflash assay

To create the TOPflash vector, pPB/Super8 TOPflash-PGKneo, the KpnI-BamHI fragment of M50 Super8 TOPFlash [46] (Addgene plasmid #12456), was subcloned into the pPBdN-Luc2-PGKneo vector. To prepare them for electroporation, C45 CTOSs were pre-treated with 5 mM EDTA/PBS for 30 min at room temperature and then mixed with pPB/Luc and the transposase expression vector pCMV-hyPBase [47]. Electroporation was performed in 2-mm gap cuvettes at 150 V for 5 ms using a TypeII NEPA21 electroporator (Nepa Gene, Chiba, Japan). TOPFlash-expressing CTOSs were established by neomycin selection. The promoter activity assay was performed using the Luciferase Assay System: E1500 (Promega) according to the manufacturer’s instructions with cell lysate prepared at each time point.

### Immunohistochemistry

CTOSs were fixed for 5 min with 10% formalin, embedded in CellMatrix I-A (Nitta Gelatin, Osaka, Japan) to condense the CTOSs, and fixed for 1 additional h. Next, gel droplets containing CTOSs were stained with Sirius red to facilitate further manipulation, and the gel droplets were embedded in paraffin. The paraffin blocks were cut into 4 μm and then used for immunostaining. Immunohistochemical analyses were performed as described previously [4]. Fluorescence images were obtained by confocal microscopy (TCS SPE, Leica Microsystems, Wetzlar, Germany).

### RNA extraction, microarray, and real-time quantitative RT-PCR

Total RNA was extracted from CTOSs with TRIzol reagent (Life Technologies) according to the manufacturer’s instructions. One microgram of total RNA was reverse-transcribed to obtain cDNA using Superscript III (Life Technologies). Microarray hybridizations were performed at Hokkaido System Science (Sapporo, Japan) using SurePrint G3 Human GE 8×60K Ver.1 (Agilent Technologies, Santa Clara, CA). The microarray slides were scanned and the gene expression profiles were analyzed at Hokkaido System Science according to the manufacturer’s protocol. Microarray data can be viewed using the NCBI Gene Expression Omnibus (GEO) accession number GSE75867.

The analysis of microarray data was initially performed in R (http://www.r-project.org/, ver. 3.1.3) with the Bioconductor packages (http://www.bioconductor.org/, ver. 3.0). The raw intensity values were subjected to a preprocessing step using the robust multi-array average (RMA) algorithm that summarizes and normalizes data into gene expression levels [48]. Each dataset was standardized using the z-score transformation method and combined for the following analysis. TIBCO Spotfire (TIBCO Software, ver. 6.5.0) was then used for hierarchical clustering by Ward’s method. To refine the regulated genes in CTOS cells, we took public datasets from the GEO database, which consisted of the “Metastasis Gene Expression Profile Predicts Recurrence and Death in Colon Cancer Patients (Moffitt Samples)” and “Metastasis Gene Expression Profile Predicts Recurrence and Death in Colon Cancer Patients (VMC Samples)” (GEO Series accession numbers GSE17536 and GSE17537, respectively [49]). Hierarchical clustering of the GSE17536 and GSE17537 datasets was performed by limiting the genes contained in the “disruption/reformation-related gene set” and the “disruption signature” data set, respectively. The datasets included in the two clusters that were classified by gene expression patterns were subjected to a survival analysis. Survival analysis was performed in R, using the “survival” packages from Bioconductor. Overall survival was measured from “overall event (death from any cause)” and “overall survival follow-up time.” The prognosis information of each sample was obtained from the “Series Matrix File” in the GEO entries. Survival curves were calculated by the Kaplan-Meier method. The log-rank test was used to assess differences in survival. *P*-values < 0.05 were considered statistically significant.

Gene set enrichment analysis (GSEA) and gene ontology (GO) analyses were performed using the default settings [50].

The quantitative PCR reactions were performed with the StepOne Real Time PCR System (Life Technologies, Carlsbad, CA) using Fast SYBR Green Master MIX. The Comparative CT Method (ΔΔCT Method) was applied according to the manufacturer’s instruction. Real Time PCR data were reported as fold-differences relative to the control sample.

The sequences of the primers that were used are as follows: human MMP7 forward: 5’cggatggtagcagtctaggg3’, reverse: 5’tgcctttaatatcatc ctgggaa3’; human IL8 forward: 5’tagcaaaattgaggccaagg3’, reverse: 5’aaaccaaggcacagtggaac3’; human ZEB1 forward: 5’tgcactgagtgtggaaaagc3’, R: 5’tggtgatgctgaaagagacg3’; human MMP1 forward: 5’tgctcatgcttttcaaccag3’, reverse: 5’ggtacatcaaagccccgata3’.

### Western blotting

Western blotting was performed as described previously [5]. The primary antibodies are described above.

### Flow cytometry

CTOSs were dissociated into single cells by treatment with 0.25% trypsin/EDTA (Life Technologies). CTOSs were dissociated into single cells 72 h after disruption for the disrupted group. The dispersed cells were stained with primary antibodies described above according to the manufacturer’s instructions. To minimize the effect of anoikis, single cells were kept on ice after dissociation until analysis, including incubation period with antibodies. Subsequently, flow cytometry was conducted using an Attune Acoustic Focusing Cytometer (Applied Biosystems, Foster City, CA, USA), and the results were analyzed using the FlowJo software (Tree Star®, Ashland, OR, USA).

### Animal studies

Animal studies were approved by the Osaka Medical Center for Cancer and Cardiovascular Diseases Institutional Animal Care and Use Committee. The mice were housed in pathogen-free conditions and supplied water *ad libitum* under controlled conditions of humidity (50% ± 10%), light (12-/12-h light/dark cycle), and temperature (25 °C). Anesthesia was induced by i.p. injection of medetomidine at 0.3 mg/kg, midazolam at 4 mg/kg, and butorphanol at 5 mg/kg. A selected number of CTOSs were injected subcutaneously in 4–6-week-old BALB/cAJcl-nu/nu mice (CLEA Japan). CTOSs were suspended in 100 μl StemPro and mixed with 100 μl of Matrigel (BD Biosciences), then injected subcutaneously using a 23G needle. CTOSs were injected into the spleen for the liver metastasis experiments and into the cecum wall for orthotopic transplantation experiments. The animals were sacrificed at the indicated time points or when they lost more than 15% of their original body weight.

### Statistical analysis

Statistical analyses were conducted using GraphPad Prism 6 (GraphPad Software, San Diego, CA, USA). Statistical significance was tested using an unpaired t-test for single comparisons and a one-way ANOVA followed by Tukey’s test for multiple comparisons. Kaplan-Meier analysis was performed using the log-rank test and contingency 2×2 tables with Fisher’s exact test. A value of *P* < 0.05 was considered to be statistically significant.

## SUPPLEMENTARY MATERIALS FIGURES AND TABLES







## Abbreviations

CRC: colorectal cancer
CTOS: cancer tissue-originated spheroid
SE: standard error
SD: standard deviation
3D: three dimensional
MAPK: mitogen-activated protein kinase
BrdU: bromodeoxyuridine
EMT: epithelial–mesenchymal transition
RT-PCR: reverse transcription-polymerase chain reaction
GSEA: gene set enrichment analysis
bFGF: basic fibroblast growth factor.
