# Supplementary material for: Promotion of malignant phenotype after disruption of the three-dimensional structure of cultured spheroids from colorectal cancer
Source: Oncotarget. 2018 Mar 23;9(22):15968–83. doi: 10.18632/oncotarget.24641 (PMC5882311; doi:10.18632/oncotarget.24641)
Supplement: Supplementary file 1 [file oncotarget-09-15968-s001.pdf]

Promotion of malignant phenotype after disruption of the three-dimensional structure of cultured spheroids from colorectal cancer

SUPPLEMENTARY MATERIALS

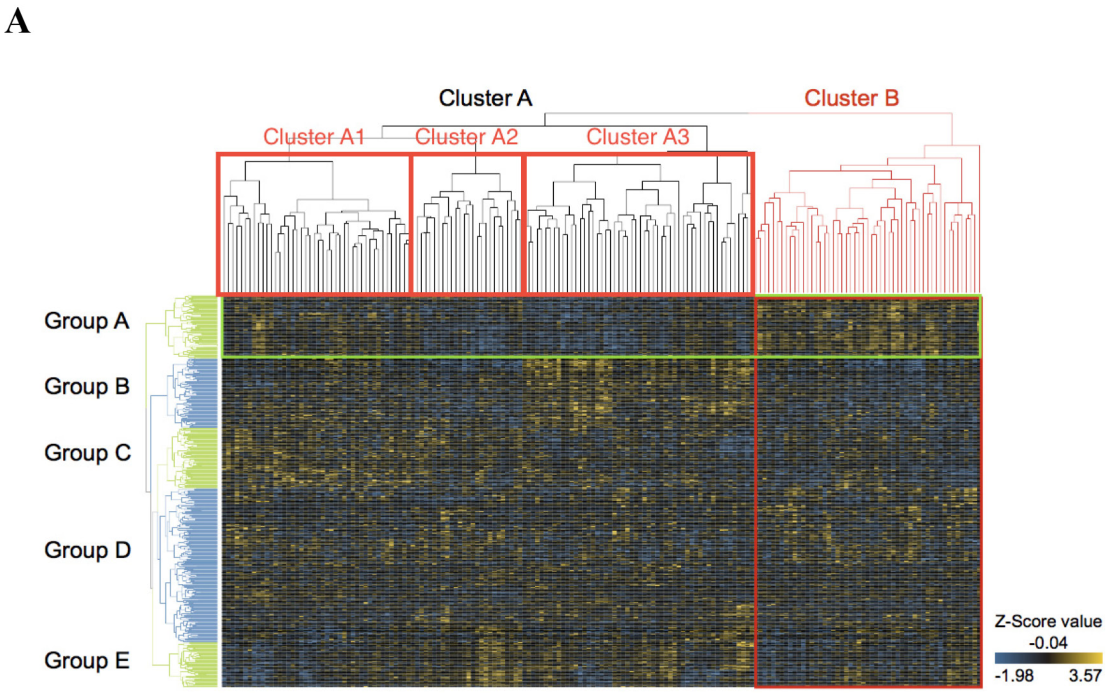

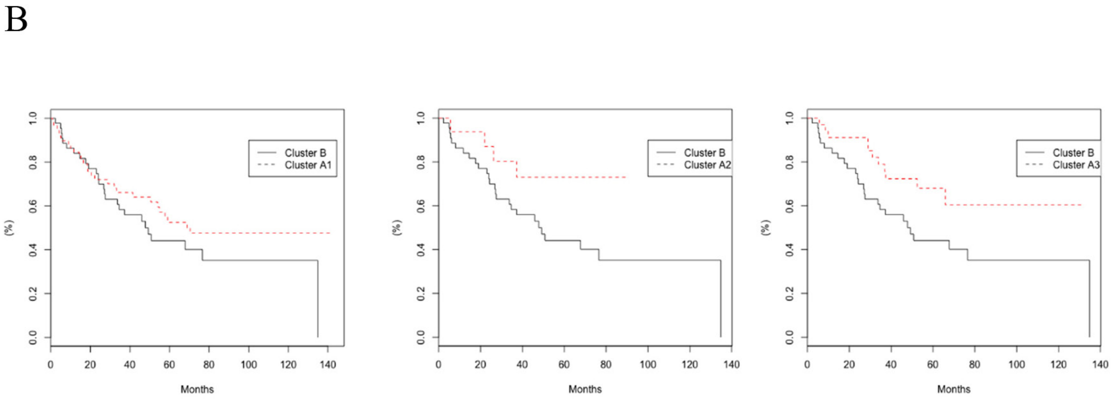

Supplementary Figure 1: (A) Cluster A in Figure 4A was subdivided into Cluster A1, a2 and A3 as designated. **(B)** Kaplan-Meier analysis of the patient's overall survival (GSE17536) according to subclusters defined in Supplementary Figure 1A. *P* =0.267, 0.014 and 0.067, from left to right respectively (log-rank test).

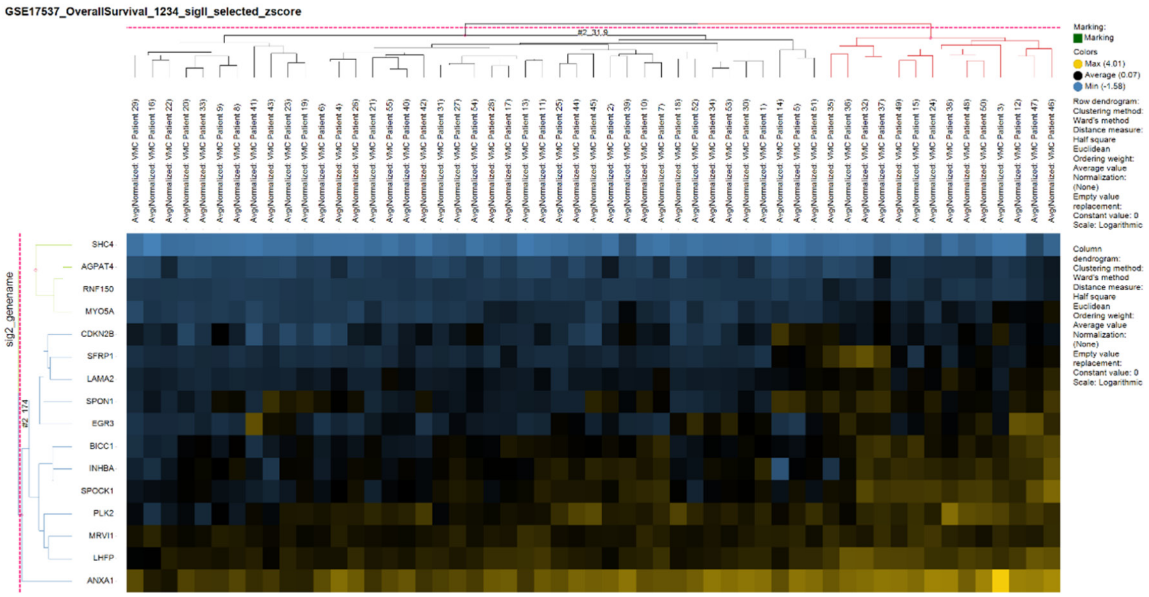

Supplementary Figure 2: The clustering heat map used for dividing patients into two groups in Figure 4E. Cases with black and red dendrogram lines are designated as Cluster A and B respectively.

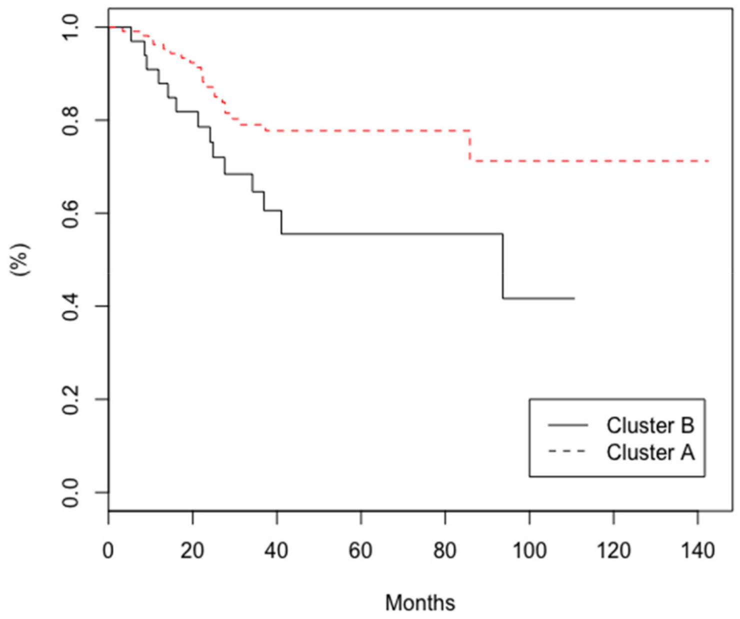

Supplementary Figure 3: Kaplan-Meier analysis of the patient's overall survival (GSE17537) according to signature group. Data lacking the information on recurrence were omitted from metadata to analyze. *P*=0.0175 (log-rank test).

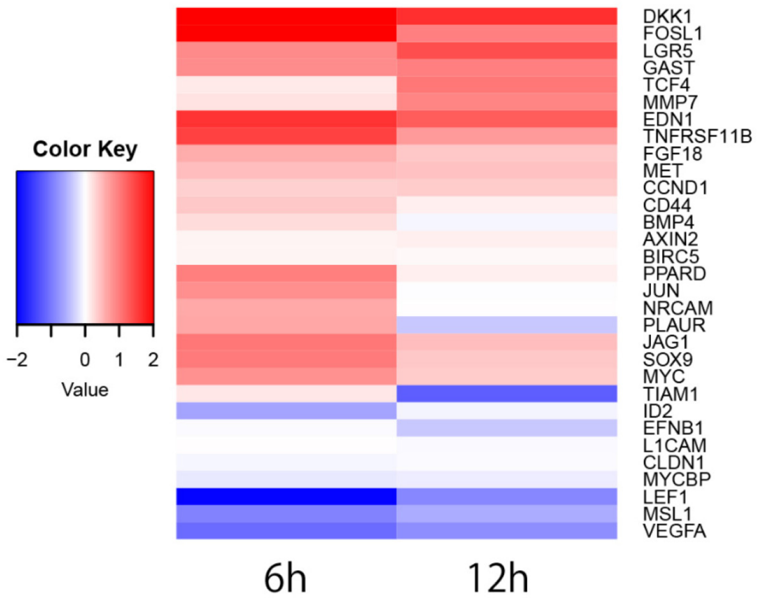

Supplementary Figure 4: Changes of the expression of known 31 WNT target genes in C45 CTOSs after disruption. Change in the expression level of each gene at each time point was normalized to pre-disruption. Fold change values in log2 (Supplementary Table 2) were visualized by R with heatmap.2.

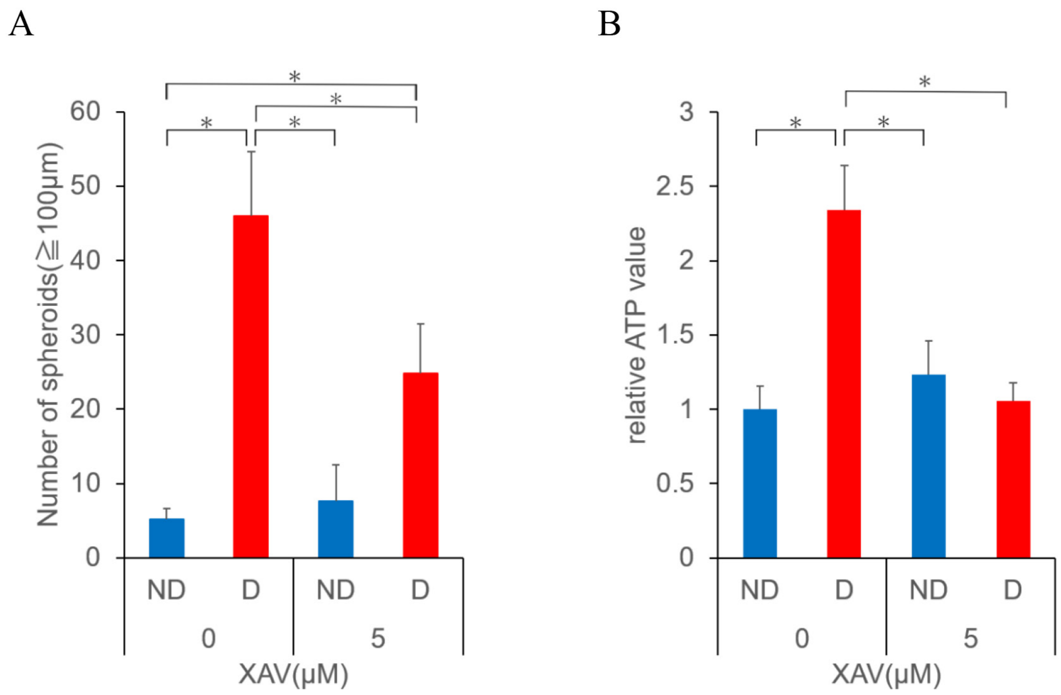

Supplementary Figure 5: (A) The number of spheroids formed from single C111 CTOS cells. Disrupted CTOSs were untreated or treated with 5 µM XAV939 for overnight, then dissociated into single cells for the spheroid formation assay. Means ± SEM are shown. N = 6. ($P = 0.00000000039$, one-way ANOVA). $^{*}P < 0.0001$; Significance according to Tukey's multiple comparison test. **(B)** Spheroids formed from single C111 CTOS cells were also evaluated by relative amount of ATP within the cultures. All cells, include spheroids smaller than 100 µm, within each well used in the experiment of Supplementary Figure 5A were subjected to ATP assay (Celltiter-Glo (Promega)). N=6, ($P = 0.0000000011$, one-way ANOVA). $^{*}P<0.0001$; Significance according to Tukey's multiple comparison test.

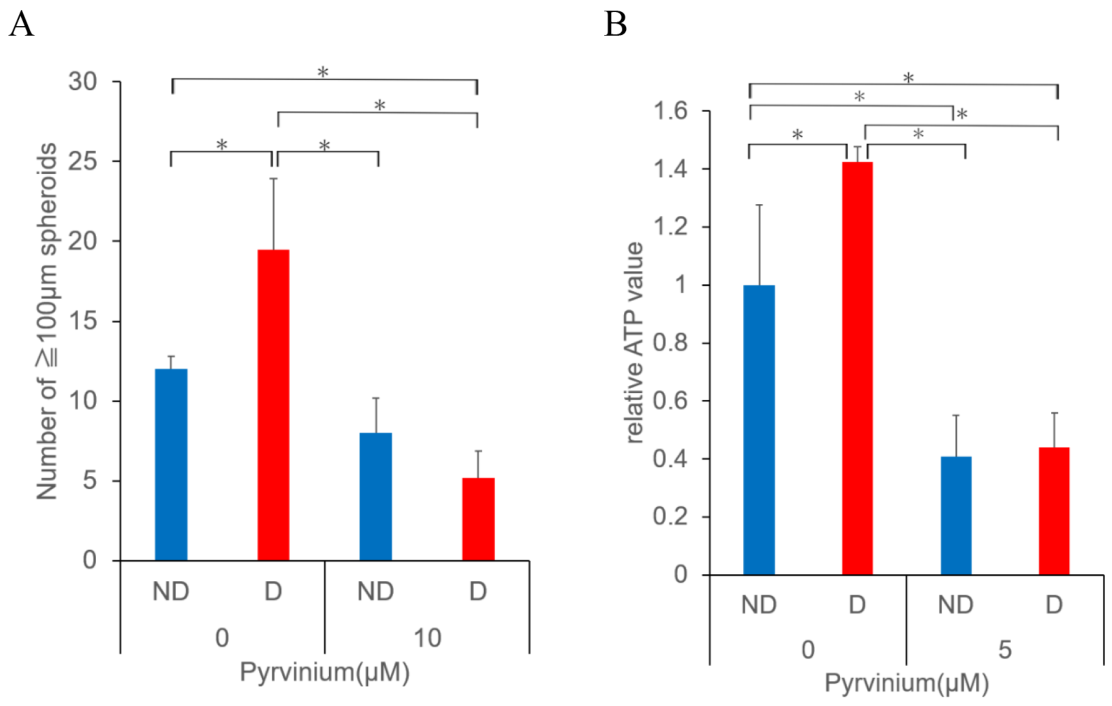

Supplementary Figure 6: **(A)** The number of spheroids formed from single C45 CTOS cells. Disrupted CTOSs were untreated or treated with 10 μM pyrvinium pomate for overnight, then dissociated into single cells for the spheroid formation assay. Means ± SEM are shown. N = 4 (P = 0.000011, one-way ANOVA). * $P < 0.001$; Significance according to Tukey's multiple comparison test. **(B)** Relative ATP value of spheroids formed from single C45 CTOS cells. Disrupted CTOSs were untreated or treated with 5 μM pyrvinium pomate for overnight, then dissociated into single cells for the assay. All cells, include spheroids smaller than 100 μm, within each well were subjected to ATP assay (Celltiter-Glo (Promega)). N = 4 wells, (P = 0.000004, one-way ANOVA). * $P<0.05$; Significance according to Tukey's multiple comparison test.

Supplementary Table 1: Disruption Signature

See Supplementary File 1

Supplementary Table 2: WNT target genes after disruption

See Supplementary File 2
