# Supplementary material for: Promotion of malignant phenotype after disruption of the three-dimensional structure of cultured spheroids from colorectal cancer
Source: Oncotarget. 2018 Mar 23;9(22):15968–83. doi: 10.18632/oncotarget.24641 (PMC5882311; doi:10.18632/oncotarget.24641)
Supplement: Supplementary file 2 [file oncotarget-09-15968-s002.docx]

Table S1 Disruption Signature

| Symbol | GenBank | Name |
| --- | --- | --- |
| ABL2 | NM_001136001 | v-abl Abelson murine leukemia viral oncogene homolog 2 (ABL2), transcript variant e |
| AGPAT4 | NM_020133 | 1-acylglycerol-3-phosphate O-acyltransferase 4 (lysophosphatidic acid acyltransferase, delta) (AGPAT4) |
| AKAP12 | NM_144497 | A kinase (PRKA) anchor protein 12 (AKAP12), transcript variant 2 |
| ALOX5AP | NM_001629 | arachidonate 5-lipoxygenase-activating protein (ALOX5AP), transcript variant 1 |
| ANKRD29 | NM_173505 | ankyrin repeat domain 29 (ANKRD29) |
| ANO1 | NM_018043 | anoctamin 1, calcium activated chloride channel (ANO1), transcript variant 1 |
| ANXA1 | NM_000700 | annexin A1 (ANXA1) |
| AOX1 | NM_001159 | aldehyde oxidase 1 (AOX1) |
| ASB2 | NM_016150 | ankyrin repeat and SOCS box containing 2 (ASB2), transcript variant 2 |
| C7orf10 | NM_024728 | chromosome 7 open reading frame 10 (C7orf10), transcript variant 4 |
| CCDC80 | NM_199511 | coiled-coil domain containing 80 (CCDC80), transcript variant 1 |
| CD59 | NM_203330 | CD59 molecule, complement regulatory protein (CD59), transcript variant 1 |
| CD86 | NM_006889 | CD86 molecule (CD86), transcript variant 2 |
| CDKN2B | NM_004936 | cyclin-dependent kinase inhibitor 2B (p15, inhibits CDK4) (CDKN2B), transcript variant 1 |
| CLIC2 | NM_001289 | chloride intracellular channel 2 (CLIC2) |
| CSF1R | NM_005211 | colony stimulating factor 1 receptor (CSF1R) |
| CSF2RA | NM_172249 | colony stimulating factor 2 receptor, alpha, low-affinity (granulocyte-macrophage) (CSF2RA), transcript variant 6 |
| CSGALNACT1 | NM_001130518 | chondroitin sulfate N-acetylgalactosaminyltransferase 1 (CSGALNACT1), transcript variant 1 |
| CTGF | NM_001901 | connective tissue growth factor (CTGF) |
| CYR61 | NM_001554 | cysteine-rich, angiogenic inducer, 61 (CYR61) |
| DSE | NM_013352 | dermatan sulfate epimerase (DSE), transcript variant 1 |
| EGR3 | NM_004430 | early growth response 3 (EGR3), transcript variant 1 |
| EMP1 | NM_001423 | epithelial membrane protein 1 (EMP1) |
| EMP3 | NM_001425 | epithelial membrane protein 3 (EMP3) |
| ETS1 | NM_005238 | v-ets erythroblastosis virus E26 oncogene homolog 1 (avian) (ETS1), transcript variant 2 |
| FAM101B | NM_182705 | family with sequence similarity 101, member B (FAM101B) |
| FLNC | NM_001458 | filamin C, gamma (FLNC), transcript variant 1 |
| FOSB | NM_006732 | FBJ murine osteosarcoma viral oncogene homolog B (FOSB), transcript variant 1 |
| FRMD4B | NM_015123 | FERM domain containing 4B (FRMD4B) |
| GLIPR1 | NM_006851 | GLI pathogenesis-related 1 (GLIPR1) |
| GREM1 | NM_001191323 | gremlin 1 (GREM1), transcript variant 2 |
| HMOX1 | NM_002133 | heme oxygenase (decycling) 1 (HMOX1) |
| HSPB8 | NM_014365 | heat shock 22kDa protein 8 (HSPB8) |
| IGFBP6 | NM_002178 | insulin-like growth factor binding protein 6 (IGFBP6) |
| IL1RN | NM_173843 | interleukin 1 receptor antagonist (IL1RN), transcript variant 4 |
| IL8 | NM_000584 | interleukin 8 (IL8) |
| INHBA | NM_002192 | inhibin, beta A (INHBA) |
| INMT | NM_001199219 | indolethylamine N-methyltransferase (INMT), transcript variant 2 |
| IQCJ-SCHIP1 | NM_001197113 | IQCJ-SCHIP1 readthrough (IQCJ-SCHIP1), transcript variant 1 |
| LAMA2 | NM_000426 | laminin, alpha 2 (LAMA2), transcript variant 1 |
| LBH | NM_030915 | limb bud and heart development homolog (mouse) (LBH) |
| LHFP | NM_005780 | lipoma HMGIC fusion partner (LHFP) |
| MEF2C | NM_002397 | myocyte enhancer factor 2C (MEF2C), transcript variant 1 |
| MMP1 | NM_002421 | matrix metallopeptidase 1 (interstitial collagenase) (MMP1), transcript variant 1 |
| MRGPRF | NM_145015 | MAS-related GPR, member F (MRGPRF), transcript variant 2 |
| MRVI1 | NM_130385 | murine retrovirus integration site 1 homolog (MRVI1), transcript variant 2 |
| MYADM | NM_001020818 | myeloid-associated differentiation marker (MYADM), transcript variant 1 |
| MYO5A | NM_000259 | myosin VA (heavy chain 12, myoxin) (MYO5A), transcript variant 1 |
| NCF2 | NM_000433 | neutrophil cytosolic factor 2 (NCF2), transcript variant 1 |
| NR3C1 | NM_001018077 | nuclear receptor subfamily 3, group C, member 1 (glucocorticoid receptor) (NR3C1), transcript variant 5 |
| NR4A3 | NM_173200 | nuclear receptor subfamily 4, group A, member 3 (NR4A3), transcript variant 3 |
| PANX1 | NM_015368 | pannexin 1 (PANX1) |
| PDE2A | NM_002599 | phosphodiesterase 2A, cGMP-stimulated (PDE2A), transcript variant 1 |
| PDGFRL | NM_006207 | platelet-derived growth factor receptor-like (PDGFRL) |
| PLAU | NM_002658 | plasminogen activator, urokinase (PLAU), transcript variant 1 |
| PLK2 | NM_006622 | polo-like kinase 2 (PLK2) |
| PMP22 | NM_000304 | peripheral myelin protein 22 (PMP22), transcript variant 1 |
| PRDM1 | NM_001198 | PR domain containing 1, with ZNF domain (PRDM1), transcript variant 1 |
| PRKCDBP | NM_145040 | protein kinase C, delta binding protein (PRKCDBP) |
| RASSF2 | NM_014737 | Ras association (RalGDS/AF-6) domain family member 2 (RASSF2), transcript variant 1 |
| RBM20 | NM_001134363 | RNA binding motif protein 20 (RBM20) |
| RCBTB2 | NM_001268 | regulator of chromosome condensation (RCC1) and BTB (POZ) domain containing protein 2 (RCBTB2) |
| RGS2 | NM_002923 | regulator of G-protein signaling 2, 24kDa (RGS2) |
| RNF150 | NM_020724 | ring finger protein 150 (RNF150) |
| RYR3 | NM_001036 | ryanodine receptor 3 (RYR3) |
| SCHIP1 | NM_014575 | schwannomin interacting protein 1 (SCHIP1), transcript variant 1 |
| SEC14L1 | NM_003003 | SEC14-like 1 (S. cerevisiae) (SEC14L1), transcript variant 1 |
| SFRP1 | NM_003012 | secreted frizzled-related protein 1 (SFRP1) |
| SGK1 | NM_005627 | serum/glucocorticoid regulated kinase 1 (SGK1), transcript variant 1 |
| SHC4 | NM_203349 | SHC (Src homology 2 domain containing) family, member 4 (SHC4) |
| SLC2A3 | NM_006931 | solute carrier family 2 (facilitated glucose transporter), member 3 (SLC2A3) |
| SOCS3 | NM_003955 | suppressor of cytokine signaling 3 (SOCS3) |
| SPOCK1 | NM_004598 | sparc/osteonectin, cwcv and kazal-like domains proteoglycan (testican) 1 (SPOCK1) |
| SPON1 | NM_006108 | spondin 1, extracellular matrix protein (SPON1) |
| ST8SIA1 | NM_003034 | ST8 alpha-N-acetyl-neuraminide alpha-2,8-sialyltransferase 1 (ST8SIA1) |
| TAGLN | NM_001001522 | transgelin (TAGLN), transcript variant 1 |
| TJP1 | NM_003257 | tight junction protein 1 (zona occludens 1) (TJP1), transcript variant 1 |
| TNC | NM_002160 | tenascin C (TNC) |
| TSPAN2 | NM_005725 | tetraspanin 2 (TSPAN2) |
| TSPAN4 | NM_001025237 | tetraspanin 4 (TSPAN4), transcript variant 1 |
| TUBB6 | NM_032525 | tubulin, beta 6 (TUBB6) |
| VIM | NM_003380 | vimentin (VIM) |
| WWTR1 | NM_015472 | WW domain containing transcription regulator 1 (WWTR1), transcript variant 1 |
| ZEB1 | NM_001128128 | zinc finger E-box binding homeobox 1 (ZEB1), transcript variant 1 |
| ZNF469 | NM_001127464 | zinc finger protein 469 (ZNF469) |
